# Considering the Cryoballoon as a Multifaceted Tool for Extensive Left Atrial Ablation in Patients with Longstanding Persistent Atrial Fibrillation

**DOI:** 10.19102/icrm.2022.130101

**Published:** 2022-01-15

**Keywords:** Ablation, atrial fibrillation, cryoablation, cryoballoon, left atrium

## Drs. Skeete and Huang discuss

Achieving sustained freedom from atrial arrhythmias following catheter ablation can be an elusive endpoint, especially in individuals with long-standing persistent atrial fibrillation (AF) (PSAF) for whom multiple mechanisms may be operant.^1^ Notwithstanding these inherent challenges, the quest for the development of catheter-based approaches for long-term maintenance of sinus rhythm in this challenging population continues. Recently, the STOP Persistent Atrial Fibrillation trial demonstrated cryoballoon (CB) ablation to be an effective and safe tool as an initial ablation approach in patients with drug-resistant PSAF.^2^ However, a perceived drawback of utilizing balloon catheter technologies in patients with PSAF has traditionally been the perception of lack of ability to perform linear ablation lesion sets or address reentrant atrial arrhythmias adequately without using radiofrequency ablation (RFA) catheters.

In this issue of the journal, Aryana et al.^1^ describe the successful use of an extensive integrated left atrial (LA) ablation approach using only a third-generation CB catheter in a patient with persistent AF who developed LA atrial flutter. In addition, the case report also reinforces the feasibility of LA appendage (LAA) electrical isolation (LAAEI) followed by implantation of an LAA occlusion device concurrently in the same procedure to avoid hazards of stasis/potential thrombosis after LAAEI in an individual for whom long-term oral anticoagulation may not be the preferred option.

### Complex ablative techniques to match a complex disease process

Central to the rationale for the approach utilized in this case was the recognition that pulmonary vein (PV) isolation (PVI) alone is often insufficient in patients with longstanding PSAF. It has been shown that the substrate for the initiation and maintenance of AF involves anatomical sites outside the PVs, such as the posterior LA wall, LAA, superior vena cava, and bundles of the LAA–left superior PV (LSPV) ridge.^3^ Furthermore, even if patients remain free from AF recurrence post-ablation, those with diseased substrate still often have a higher proclivity for developing reentrant atrial arrhythmias, which must be addressed with additional linear lesion sets. Thus, an argument could be made that upfront extensive ablation approaches may be beneficial in some cases to debulk and potentially address problematic substrate and limit the “real estate” available for the perpetuation of reentrant arrhythmias.

### Revisiting the role of the posterior left atrial wall in persistent atrial fibrillation

The posterior wall of the LA has been implicated in the initiation and maintenance of PSAF. In a meta-analysis of 17 studies by Thiyagarajah et al.^4^ the acute procedural success rate of PVI + posterior wall isolation (PWI) was 94.1% (confidence interval [CI], 87.2%–99.3%). Among patients with PSAF, 12-month freedom from atrial arrhythmias following a single procedure was 61.9% (95% CI, 54.2%–70.8%), which was similar to the effectiveness of PVI only. Notably, two atrioesophageal fistulas occurred (n = 1,667), both cases in the RF–PWI group. A subsequent randomized controlled trial (RCT) by Lee et al.^5^ also found that a PWI strategy did not lead to a significant difference in freedom from AF (posterior wall box isolation vs. circumferential PVI, 55.9% vs. circumferential PVI; P = .522) at 16 months. However, an RCT by Aryana et al.^6^ comparing PVI alone to PVI + PWI in patients with longstanding PSAF demonstrated a significant reduction in recurrence of AF at one year (25.5% vs. 45.5%; P = .028) using CB ablation with an adjunctive RFA approach. A large multicenter study—Left Atrial Posterior Wall and Posterior Vein Isolation Using Cryoballoon for Treatment of Persistent Atrial Fibrillation (PIVoTAL-IDE)—is currently underway to further evaluate the efficacy and safety of this approach.^7^

### Left atrial appendage isolation—what does the evidence suggest?

Another strategy employed in this case report by Aryana et al.^1^ was LAAEI using an ostial CB ablation approach. Previously Di Biase et al.^8^ examined 987 patients (71% with non-paroxysmal AF) and observed that, in 27% of patients referred for a redo ablation, the LAA appeared to be the source of drivers for AF. When LAAEI was performed, the rate of AF recurrence was 15%, compared to 74% in patients who did not receive ablation and 68% in patients undergoing only focal ablation (P < .001). These findings were further supported in the Left Atrial Appendage Isolation in Patients with Longstanding Persistent Atrial Fibrillation Undergoing Catheter Ablation (BELIEF) trial.^9^ However, some concerns have been raised about the risk of post-procedural LAA thrombus, which was detected in 23.2% of patients who underwent LAAEI versus 2.2% in the control arm (P < .001) in a prospective study despite appropriate anticoagulation.^10^ A subsequent systematic review and meta-analysis including 2,336 patients in 9 studies by Romero et al.^11^ found no significant difference in the rates of cerebral thromboembolism with LAAEI versus standard LA ablation (3% vs. 1.6%; P = .29).^9^ Nonetheless, in the case presented by Aryana et al.,^1^ an LAA occlusion device was implanted in the same procedure to mitigate the risk of post-procedural thromboembolism.

Although concurrent RF PVI and LAAO implantation has been associated with the incidence of increased or new significant (>5 mm) peri-device leak on post-procedural imaging,^12^ several prospective studies examining combined CB PVI and LAAO seem to suggest a low incidence of developing significant peri-device leaks post-procedure.^13,14^ Thus far, only a single study^15^ (n = 22) has prospectively studied the LAAEI approach versus RF PVI only and found higher freedom from AF in the LAAEI group (95% vs. 63%; P = .036) and an LAAEI time of 33.5 ± 27.7 minutes. In the cohort, 5% of patients developed peri-device leaks greater than 5 mm on 45-day transesophageal echocardiography imaging. Whether the CB technique can be utilized as a more efficient approach for LAAEI with perhaps a lower risk of peri-device leak after device implant may be an important area of future study.

### Clinical implications and further thoughts

The case presented by Aryana et al.^1^ demonstrates the feasibility and importance of developing innovative techniques for single-modality ablation, especially as recent trends in catheter technology development have focused more so on improving acute procedural endpoints mainly for PVI. As increasing financial constraints limit the practicality of mixing energy sources in the same procedure, development of single-modality techniques for extensive LA ablation may become of increasing importance. Patients with longstanding PSAF have traditionally fared poorly with ablation strategies. Looking forward, larger clinical studies will be needed to validate the generalizability of the approach used in this case study given the higher operator learning curve expected to master techniques in this report.

Intriguing is that utilization of a CB approach for the creation of linear lesion sets may have the potential to significantly shorten the procedure time needed for debulking due to a wider electrical effect on tissue than that with point-by-point RFA. At the same time, it may be more difficult to discern regions with sustained reversible injury versus areas destined for irreversible necrosis following cryoenergy applications as durable lesion formation is most likely in areas in direct contact with the balloon surface (area of maximal heat transfer).^16^ Also, it may be more difficult to judge proper placement of CB lesions when attempting linear ablation compared to point-by-point RFA where catheter contact and location can be accurately assessed in real time due to integration with 3-dimensional mapping systems. In this instance, the authors address this limitation by utilizing intracardiac echocardiography (ICE) visualization before cryoenergy applications. Finally, heat sink effects on energy transfer, which affect other thermal energy sources similarly, exist during cryoenergy delivery and may attenuate the depth of tissue injury in thicker areas adjacent to the coronary sinus or LAA–LSPV ridge (vein of Marshall) in a way that could affect the durability of LAA isolation or ability to address certain circuits of perimetral flutter (albeit also challenging for RF).^17^

In summary, the case study presented by Aryana et al. is an elegant and convincing initial case demonstration that, at experienced centers, the CB catheter is a potentially capable multifaceted tool when extensive LA ablation is required in patients with PSAF. This case report also emphasizes the importance of consideration of LAA placement to mitigate the risk of thromboembolism after LAAEI.

Jamario R. Skeete, MD^1^ and Henry D. Huang, MD, FHRS (henry_d_huang@rush.edu)^1^

^1^Rush University Medical Center, Chicago, IL, USA

The authors report no conflicts of interest for the published content.

References1.AryanaALafortunePDi BiaseLNataleACryoballoon ablation of the pulmonary veins, the posterior wall, the left atrial appendage, and the mitral isthmus guided by intracardiac echocardiography and three-dimensional mappingJ Innov Cardiac Rhythm Manage20211314865487010.19102/icrm.2022.130105PMC8812479351272412.IwasakiYKNishidaKKatoTNattelSAtrial fibrillation pathophysiology: implications for managementCirculation20111242022642274[CrossRef]
[PubMed]2208314810.1161/CIRCULATIONAHA.111.0198933.SuWWReddyVYBhasinKCryoballoon ablation of pulmonary veins for persistent atrial fibrillation: results from the multicenter STOP persistent AF trialHeart Rhythm2020171118411847[CrossRef]
[PubMed]3259015110.1016/j.hrthm.2020.06.0204.ZhaoYDi BiaseLTrivediCImportance of non-pulmonary vein triggers ablation to achieve long-term freedom from paroxysmal atrial fibrillation in patients with low ejection fractionHeart Rhythm2016131141149[CrossRef]
[PubMed]2630471310.1016/j.hrthm.2015.08.0295.ThiyagarajahAKadhimKLauDHFeasibility, safety, and efficacy of posterior wall isolation during atrial fibrillation ablationCirc Arrhythm Electrophysiol2019128e007005[CrossRef]
[PubMed]3140185310.1161/CIRCEP.118.0070056.LeeJMShimJParkJThe electrical isolation of the left atrial posterior wall in catheter ablation of persistent atrial fibrillationJACC Clin Electrophysiol201951112531261[CrossRef]
[PubMed]3175342910.1016/j.jacep.2019.08.0217.AryanaAAllenSLPujaraDKConcomitant pulmonary vein and posterior wall isolation using cryoballoon with adjunct radiofrequency in persistent atrial fibrillationJACC Clin Electrophysiol202172187196[CrossRef]
[PubMed]3360239910.1016/j.jacep.2020.08.0168.AryanaAPujaraDKAllenSLLeft atrial posterior wall isolation in conjunction with pulmonary vein isolation using cryoballoon for treatment of persistent atrial fibrillation (PIVoTAL): study rationale and designJ Interv Card Electrophysiol2021621187198[CrossRef]
[PubMed]3300964510.1007/s10840-020-00885-wPMC82107449.Di BiaseLBurkhardtJDMohantyPLeft atrial appendage: an underrecognized trigger site of atrial fibrillationCirculation20101222109118[CrossRef]
[PubMed]2060612010.1161/CIRCULATIONAHA.109.92890310.Di BiaseLBurkhardtJDMohantyPLeft atrial appendage isolation in patients with longstanding persistent AF undergoing catheter ablation: BELIEF trialJ Am Coll Cardiol2016681819291940[CrossRef]
[PubMed]2778884710.1016/j.jacc.2016.07.77011.HeegerCHRilligAGeislerDLeft atrial appendage isolation in patients not responding to pulmonary vein isolationCirculation20191395712715[CrossRef]
[PubMed]3068941610.1161/CIRCULATIONAHA.118.03745112.RomeroJGabrMPatelKEfficacy and safety of left atrial appendage electrical isolation during catheter ablation of atrial fibrillation: an updated meta-analysisEuropace2021232226237[CrossRef]
[PubMed]3332497810.1093/europace/euaa26613.PhillipsKPWalkerDTHumphriesJACombined catheter ablation for atrial fibrillation and Watchman^®^ left atrial appendage occlusion procedures: five-year experienceJ Arrhythm2016322119126[CrossRef]
[PubMed]2709219310.1016/j.joa.2015.11.001PMC482357714.FassiniGContiSMoltrasioMConcomitant cryoballoon ablation and percutaneous closure of left atrial appendage in patients with atrial fibrillationEuropace2016181117051710[CrossRef]
[PubMed]2740262310.1093/europace/euw00715.RenZZhangJWangSTwo-year outcome from combining cryoballoon ablation and left atrial appendage closure: CLACBAC studyFront Cardiovasc Med20217610537[CrossRef]
[PubMed]3350599410.3389/fcvm.2020.610537PMC782921316.PanikkerSJarmanJWEVirmaniRLeft atrial appendage electrical isolation and concomitant device occlusion to treat persistent atrial fibrillationCirc Arrhythm Electrophysiol201697e003710[CrossRef]
[PubMed]2740660210.1161/CIRCEP.115.00371017.TakamiMMisiriJLehmannHISpatial and time-course thermodynamics during pulmonary vein isolation using the second-generation cryoballoon in a canine in vivo modelCirc Arrhythm Electrophysiol201581186192[CrossRef]
[PubMed]2553252910.1161/CIRCEP.114.00213718.JonesDLGuiraudonGMSkanesACGuiraudonCMAnatomical pitfalls during encircling cryoablation of the left atrium for atrial fibrillation therapy in the pigJ Interv Card Electrophysiol2008213187193[CrossRef]
[PubMed]1832445910.1007/s10840-008-9205-6

## Dr. Tondo examines

Aryana et al.^1^ report a case presentation of a patient with persistent AF treated by combining PVI, PWI, LAA isolation, mitral isthmus ablation by CB ablation, and eventually LAA mechanical closure with an endocardial device. This is a clear example of how feasible and safe CB (and thus cryoenergy) is, even outside of the PVs, as a more aggressive approach for restoring and maintaining regular sinus rhythm in a patient with advanced atrial arrhythmia.

There are reasons why PWI of the LA (LPWI) needs to be included when ablation of persistent AF is planned. First, LPW shares the embryological origin of the PVs, which implies that the two structures are intertwined, potentially favoring creation of different and complex circuits. In this respect, it is worth mentioning that, as the LA develops, the PVs represent an outgrowth tissue of the LPW. This explains why the embryologic origin of the LPW and PVs can provide the anatomic basis for the development of atrial arrhythmias and, in particular, of AF.^2,3^ Furthermore, atrial myocytes in LPW have a higher incidence of delayed afterdepolarizations and larger late sodium currents in addition to larger intracellular Ca transients and sarcoplasmic reticulum Ca contents. This can promote a greater propensity to arrhythmogenic behavior and lead to distinctive electrophysiological properties that may contribute to the pathophysiology of AF.^4^

The LPW in patients with persistent AF is an ideal anatomic location for significant atrial remodeling, including fibrosis and lymphomononuclear infiltration.^5,6^ Moreover, spontaneous trigger activity and so-called rotors have been previously reported in persistent AF patients.^7,8^ Therefore, since secondary triggers and more complex atrial circuitries take place in the LPW, it appears reasonable that their elimination may be a crucial adjuvant strategy for providing a better clinical outcome. Aryana et al.^1^ elegantly describe how to safely maneuver the CB in the LA so as to properly cover the entire LPW. As the ablative techniques and the technology in the field evolve, how to perform isolation of the LPW remains a very debatable, controversial issue. Currently, the majority of operators use point-by-point radiofrequency current for LPWI by performing a roof line and a floor line (two lines connecting the contralateral PV-encircling lesions). In addition, mitral isthmus ablation can be added. Several issues need to be addressed when linear lesions in the LA posterior wall are taken into account. There is an inherent technical difficulty in achieving a successful electrical LPW isolation by a set of linear radiofrequency lesions due to the complex anatomical architecture of the atrial musculature. Again, even if conduction block along the lines is achieved during the procedure, one cannot rule out the occurrence of gaps over time and, thus, dormant conduction may take place during the follow-up. Aryana et al.,^1^ by adopting a CB technique, have demonstrated the feasibility of creating an effective homogenization of the LPW. It looks like the specific design of CB facilitates a more homogenous tissue ablation.

Moreover, what makes this clinical case unique is the additional LAA isolation and, then, the mitral isthmus ablation for the occurring mitral-dependent atrial flutter following LPWI. Since the LAA can harbor secondary, non-PV triggers responsible for recurrent and self-sustained AF, the choice to electrically isolate the LAA appears reasonable, especially when the mechanical closure of this anatomical structure is planned (as in their patient with previous major gastrointestinal bleeding). Concomitant CB ablation and LAA closure have already been described by our group,^9^ showing the feasibility, effectiveness, and safety of such an approach. Electrical LAA isolation is surely to be acknowledged as an adjunctive maneuver than can improve the clinical outcome of persistent AF ablation. What appears very promising is how fast is LAA isolation promoted by CB can occur as compared to the long time required when RF energy is applied.

The authors of the present case are known as masters of the CB technique and, thus, mitral isthmus line creation can also be safely and effectively performed. Moreover, this clinical case highlights the role of ICE in guiding the 3-dimensional reconstruction of the LA and also showing the position of CB when it is maneuvered toward the several anatomic ablation sites in the LPW and the entrance of the LAA. Also, the ICE probe, when moved transeptally to the LA, can offer excellent views of the LAA and, thus, guide the implant of an occluder.

In summary, the overall information that we collect from this clinical report notifies us that the LPW plays a pivotal role in the maintenance of AF and should be included in the ablative approach of patients with persistent AF. Probably, in order to obtain an effective and durable LPWI, the scheme of lesions should be properly established so as to reduce the likelihood of electrical reconnection of the posterior wall. The result achieved by Aryana et al. also reinforces the concept that a more aggressive approach needs to be carried out in patients with more advanced AF, adding LAA isolation followed by mechanical closure with an endocardial occluder.

Even with the inherent limitations of a single clinical case, we should applaud Aryana et al. for drawing our attention to the pivotal role of the LPW in the initiation and maintenance of AF and therefore underlying the value of walking out of the PVs toward the posterior wall. Achieving proven isolation of LPW is essential along with the abolition of sources of non-PV triggers (such as the LAA) in order to promote additional benefits over PVI alone in the treatment of persistent AF.

Claudio Tondo, MD, PhD, FESC, FHRS (claudio.tondo@cardiologicomonzino.it)^1^

^1^Centro Cardiologico Monzino, Heart Rhythm Center, Department of Clinical Electrophysiology and Cardiac Pacing, Department of Biochemical, Surgical and Dentist Sciences, University of Milan, Milan, Italy

The author reports no conflicts of interest for the published content.
